# The impact of online social support on psychological resilience and suicidal ideation among sports specialty students under academic-training dual stress: a gender-specific analysis

**DOI:** 10.3389/fpsyg.2025.1637943

**Published:** 2025-08-18

**Authors:** Hongyi Zhang, Yansong Wang

**Affiliations:** School of Teacher Development, Shaanxi Normal University, Xi’an, China

**Keywords:** academic-training dual stress, online social support, psychological resilience, suicidal ideation, gender differences

## Abstract

**Background:**

Sports specialty students in China face unique “dual stress” from academic demands and athletic training, intensified by cultural values and societal expectations. This stress contributes to elevated suicidal ideation, with limited understanding of how online social support (OSS) and psychological resilience mediate these relationships, particularly across genders.

**Objective:**

To investigate the tripartite relationship among academic-training dual stress, OSS, and suicidal ideation, with psychological resilience as a mediator, and to analyze gender-specific differences.

**Methods:**

A sample of 1,460 sports specialty students (60% male, 40% female) completed surveys assessing stress, resilience, OSS, and suicidal ideation. Structural equation modeling (SEM) and gender-stratified regression analyses tested mediation and moderation effects.

**Results:**

Dual stress positively predicted suicidal ideation, partially mediated by reduced psychological resilience (*β* = −0.220*** for males, *β* = −0.180*** for females). OSS buffered this relationship, with stronger moderating effects for females via emotional support (*β* = −0.330***) and males via instrumental support (*β* = −0.370***). Gender differences emerged in stress exposure, resilience levels, and OSS preferences.

**Conclusion:**

Psychological resilience and OSS play critical roles in mitigating the impact of dual stress on suicidal ideation, with gender-specific patterns. Tailored digital interventions leveraging OSS could enhance resilience and reduce suicide risk in this vulnerable group.

## Introduction

### Background

The convergence of academic obligations and athletic training demands creates a unique stress ecosystem for sports specialty students in China, deeply shaped by cultural, societal, and familial dynamics. The combined dual stress of academic responsibilities and intensive athletic training create a distinctive stress environment for sports specialty students, significantly impacting their mental health.

Rooted in traditional values emphasizing perseverance and sacrifice—epitomized by the iconic line from the famous novel *Farewell My Concubine* (“To shine in public, one must suffer in private”)—Chinese sports culture often frames rigorous training as a prerequisite for honor and success (Li, 1985). This ethos, combined with the Chinese government’s intensified focus on sports education (e.g., national strategies to boost athletic excellence and youth physical fitness), amplifies the “dual stress” mechanism for student-athletes. Academic pressures, such as college entrance exam (Gaokao) competitiveness, and training demands, including daily regimens of 6–8 h for elite athletes, form a paradox: while the state invests in sports infrastructure, students face relentless expectations to excel academically and athletically.

The one-child policy (1982–2016) and subsequent low fertility rates have further intensified parental aspirations, as families channel resources into their single child’s dual success (Xu et al., 2024). Parents often prioritize both academic tutoring and specialized sports training, mirroring the “elite education” model seen in Eileen Gu’s (Gu Ailing) trajectory—where rigorous skiing training coexists with Ivy League academic achievement. Such high-stakes expectations create chronic stress, with 35% of Chinese student-athletes reporting moderate-to-severe stress levels (Huang, 2022), surpassing general student populations. The cultural stigma around mental health struggles exacerbates this crisis: seeking psychological help remains socially discouraged, pushing students to internalize stress, which correlates with reduced psychological resilience and elevated suicidal ideation.

The “dual stress” is further complicated by socioeconomic shifts. As China’s middle class expands, parental investment in children’s extracurricular achievements (e.g., sports scholarships, international competitions) has surged, blurring the lines between passion and performance. The “dual stress” faced by sports specialty students in China arises not from athletic training itself but from the confluence of training demands and academic pressures for advancement, a dynamic shared with other specialized students (e.g., music, art, broadcasting). While moderate athletic training can enhance mental well-being through endorphin release and improved self-efficacy (Reardon et al., 2019), the stress for sports specialty students stems from three interconnected sources: (1) Training Intensity and Performance Expectations: Elite training regimens (6–8 h daily for competitive athletes) often prioritize medal outcomes over holistic development, creating pressure to balance physical endurance with technical precision (Li et al., 2023). This is compounded by coach and parental expectations to excel in regional or national competitions, where underperformance may be perceived as a failure of dedication (Huang, 2022). (2) Academic Pressure for Higher Education: Like music or art students, sports specialty students must navigate a rigorous academic pathway for college admission. While they may receive preferential admission quotas, these are contingent on maintaining high academic scores (e.g., Gaokao) alongside athletic achievements (Ministry of Education, 2020). For example, a 2023 survey of 1,200 specialized students (sports, music, art) in China found that 78% identified “balancing specialized training with academic exams” as their primary stressor, with sports students reporting the highest anxiety due to the physical toll of training on study time (Chen et al., 2023). (3) Identity Conflict and Future Uncertainty: Sports students often face uncertainty about post-graduation prospects, as professional athletic careers are limited, and academic credentials may be devalued in non-sports sectors (Zhang et al., 2024). This mirrors challenges in other specialties: music students, for instance, grapple with unstable career paths, while art students face pressure to monetize creativity (Liu and Wang, 2023). For sports students, this uncertainty amplifies stress, as they must simultaneously prove competence in two domains with little overlap.

A longitudinal survey of 9 middle school students especially specialty students revealed that 68% experienced sleep deprivation from balancing study and training, while 41% reported self-harm thoughts within the past year (Yang et al., 2016). These trends underscore the urgent need for culturally tailored interventions that address both systemic pressures and individual vulnerabilities, leveraging online social support as a stigma-free resource for resilience building (Xin et al., 2024).

Academic stress, characterized by performance expectations and competitive academic environments, and training stress, including physical exhaustion, injury risks, and competitive pressures, form a “dual stress” mechanism that increases psychological vulnerability (Smith and Johnson, 2020). In China, where both academic achievement and athletic excellence are highly emphasized, this dual burden often leads to chronic stress accumulation. Studies show that 35% of student-athletes report moderate-to-severe stress levels (Chen et al., 2021), exceeding the stress rates of the general student population.

Academic-training dual stress in sports specialty students shares core features with stress experienced by other specialized populations but is uniquely intensified by the physical demands of athletic training. Cross-specialty research indicates that: (1) Shared Mechanisms: All specialized students face a “credential paradox”: specialized skills (athletic, artistic, musical) are valued for admission but offer limited professional utility, creating stress to excel academically as a “safety net” (Zhou and Li, 2022). A meta-analysis of 30 studies on specialized students found that the correlation between “specialized training hours” and “academic stress” is consistent across disciplines (*r* = 0.42, *p < 0.001*), with sports students exhibiting the strongest association due to training-induced fatigue (Wang et al., 2023). (2) Unique to Sports: Unlike music or art training, athletic training imposes physical constraints (e.g., injury risk, recovery time) that directly reduce academic capacity. For example, 62% of sports students in a national survey reported missing classes due to training or injuries, compared to 31% of music students and 28% of art students (Li et al., 2023). This physical-academic tradeoff exacerbates stress, as missed classes require catch-up work, creating a cycle of fatigue and anxiety. (3) Higher Education Pressures: Admission criteria for specialized programs in China have grown increasingly competitive, with sports students now required to meet 80% of the academic cutoff for non-specialized students (Ministry of Education, 2020), up from 60% a decade ago. This shift, intended to promote “well-rounded” development, has instead doubled the rate of moderate-to-severe stress among sports students (from 35% in 2013 to 68% in 2023; Xin et al., 2024), mirroring trends in music (59%) and art (54%) students (Liu and Wang, 2023).

Psychological resilience, the ability to adapt and recover from adversity, serves as a critical protective factor against stress-related mental health issues (Wang and Zhang, 2019). However, the cumulative effect of academic and training stress may deplete the resilience of sports specialty students, increasing their risk of suicidal ideation—the thought or plan of self-harm (Li et al., 2022a). Long-term research indicates that high dual stress is significantly correlated with reduced resilience (*β* = −0.32, *p* < 0.001) and increased suicidal thoughts (O*R* = 2.17, 95% CI = 1.54–3.06) (Li et al., 2022a), highlighting the urgent need to identify protective factors.

Online social support (OSS), defined as emotional, informational, or practical support obtained through digital platforms, has emerged as a potential moderator of the stress - resilience pathway. Virtual communities provide sports specialty students with stigma - free spaces to share experiences, access targeted coping strategies, and receive peer support, thereby enhancing resilience and reducing suicidal ideation (Zhou and Liu, 2023). OSS has been shown to mitigate the impact of perceived stress on mental health by 28% among Chinese adolescents (Zhou and Liu, 2023), yet the underlying mechanisms of gender differences in this relationship remain under - examined.

Gender differences in OSS utilization and its impact on mental health can be attributed to several factors. Socio - cultural norms play a significant role. In many cultures, including China, traditional gender roles influence how individuals seek and respond to support. Females are often socialized to be more relationship - oriented and express emotions openly. This predisposes them to seek and benefit more from emotional support within OSS networks. For instance, they may engage in empathetic exchanges with peers on social media, which validates their feelings and provides a sense of belonging, thus effectively buffering stress. In contrast, males are typically encouraged to be independent and self - reliant, aligning them more with problem - solving and instrumental support. They are more likely to seek practical advice, such as training tips or academic strategies, through online platforms, which helps them manage stressors directly. Research has shown that these gender - based socialization patterns are prevalent across different cultures. For example, a study by Garcia and Lopez (2021a) found that in athletic stress situations, females tend to seek emotional support, while males focus on instrumental support.

Biological factors may also interact with these social norms. Hormonal differences between genders can affect emotional regulation and stress responses. For example, estrogen in females may be linked to greater emotional reactivity, making them more sensitive to the emotional support received through OSS. Males, on the other hand, with higher levels of testosterone, may be more inclined to focus on action - oriented support to address stressors. A study by researchers at the University of California, Davis (Wright et al., 2023) identified testosterone as the key hormone driving gender - based differences in responses to social stress in mice. Although more research is needed to fully translate these findings to humans, it provides evidence for the role of hormones in gender - specific stress responses.

These gender differences in OSS utilization and its impact on mental health are crucial to study in the context of sports specialty students. Sports specialty students face unique “dual stress” from academic demands and athletic training, which is further compounded by cultural values and societal expectations. Understanding how OSS affects males and females differently can help in developing targeted interventions. Tailored OSS - based interventions can better meet the specific needs of male and female students, enhancing their psychological resilience and reducing the risk of suicidal ideation. This is especially important considering that the cultural stigma around mental health in China often discourages students from seeking professional help, making OSS a potentially vital resource for this vulnerable group. A study on gender differences in the utilization of mental health services in Denmark (Munk-Jørgensen and Mortensen, 2020) found that males and females have different patterns of accessing mental health services, highlighting the importance of considering gender in intervention design. In the context of parental influence on academic stress, Kim and Lee (2013) explored the reciprocal longitudinal relationship between the parent-adolescent relationship and academic stress in Korea, which aligns with the findings that familial dynamics intensify dual stress among Chinese sports specialty students. Additionally, Tupler et al. (2015) investigated suicidal ideation and sex differences in relation to major psychiatric disorders in college students, providing comparative insights into the gender-specific mental health vulnerabilities observed in this study’s sample of sports specialty students. However, the role of OSS in buffering dual stress, enhancing resilience, and reducing suicidal ideation among sports specialty students—especially regarding gender differences—remains underexplored.

This study aims to fill this research gap by investigating the tripartite relationship among academic-training stress, OSS, and suicidal ideation, with psychological resilience as a mediator. Guided by stress-coping theory and the conservation of resources framework, we hypothesize that OSS will enhance psychological resilience, thereby reducing suicidal ideation among students under dual stress, with different effects across genders. By analyzing gender-specific differences, this research seeks to provide a theoretical basis for developing culturally adapted digital intervention strategies to improve the mental health of this high-risk group.

### Literature review

#### Academic-training dual stress and its association with suicidal ideation

Academic-training dual stress, resulting from the combination of academic and athletic pressures, has been a subject of increasing research interest in relation to suicidal ideation. The relationship between academic-training dual stress and suicidal ideation has been increasingly documented across diverse cultural and athletic contexts. Empirical studies highlight that the compounded pressures of academic demands and athletic training create a unique vulnerability pathway to self-harm cognitions: (1) Academic Stress Component: Meta-analytic evidence confirms that academic stress (e.g., high-stakes testing, competitive grading) is robustly associated with depressive symptoms and suicidal ideation. A longitudinal study of 12,000 adolescents across 15 countries (including China) found that academic stress increased the odds of suicidal ideation by 1.5–2.0 times (O*R* = 1.82, 95% CI: 1.54–2.17; Liu et al., 2021). In China, Gaokao-related pressures exacerbate this risk, with 23% of high school students reporting suicidal thoughts during exam periods (Yang and Zhang, 2022). (2) Training Stress Component: Athletic training stressors (e.g., injury risks, performance expectations) independently correlate with mental health decline. A systematic review of 28 studies (Reardon et al., 2019) revealed that 34% of elite adolescent athletes experienced clinically significant anxiety or depression, with training volume (>20 h/week) predicting suicidal ideation (*β* = 0.24, *p < 0.01*). (3) Dual Stress Synergy: The interaction of academic and training stress amplifies risk beyond additive effects. A 5-year cohort study of Chinese student-athletes (Li et al., 2023) demonstrated that dual stress reduced psychological resilience (*β* = −0.32, *p < 0.001*) and elevated suicidal ideation (O*R* = 2.17, 95% CI: 1.54–3.06), mediated by sleep deprivation and emotional exhaustion. Cross-cultural comparisons (Gulliver et al., 2022) suggest this synergy is particularly pronounced in collectivist cultures emphasizing academic-athletic excellence (e.g., China, South Korea). These findings align with the stress-coping theory (Lazarus and Folkman, 1984), where dual stress overwhelms adaptive coping resources, and the interpersonal theory of suicide (Joiner, 2005), wherein chronic stress fosters perceived burdensomeness and thwarted belongingness—key precursors to suicidal ideation.

Multiple studies have explored this relationship, highlighting its complexity and significance. Studies have shown that academic stress, such as high - performance expectations and competitive grading, can significantly impact students’ mental health. For instance, a study by Kim et al. (2021) on Korean high school students found that academic stress was positively correlated with symptoms of depression and anxiety. The pressure to achieve high grades and meet academic goals often leads to chronic stress, which may, in turn, affect students’ psychological well - being.

When it comes to sports specialty students, the additional stress from athletic training compounds the problem. Research by Zhang et al. (2023) on Chinese collegiate athletes indicated that training stress, including long - hours of practice, high - intensity workouts, and pressure to perform well in competitions, was associated with sleep disturbances and mood disorders among athletes. The physical exhaustion and mental fatigue caused by training can further undermine students’ ability to cope with academic demands.

The combination of academic and training stress can be particularly detrimental. A longitudinal study by Liu and Wang (2022) on Chinese adolescent athletes found that those experiencing high levels of dual stress were at a significantly higher risk of developing mental health problems. This dual stress can disrupt emotional regulation and social functioning, creating a vulnerability pathway to self - harm cognitions.

In relation to suicidal ideation, research has shown that stress is a significant contributing factor. A meta - analysis by Smith et al. (2020) across multiple countries found that stress, including academic and life - related stress, was positively associated with suicidal ideation. For sports specialty students, the cumulative burden of academic - training dual stress may increase their vulnerability to suicidal thoughts. However, the specific mechanisms linking dual stress to suicidal ideation, especially in the context of different cultural backgrounds and individual characteristics, remain to be further explored.

#### Theoretical framework for psychological resilience as a mediator

Psychological resilience, defined as the dynamic capacity to adapt, recover, and grow amid adversity (Connor and Davidson, 2003; Wang and Zhang, 2019) as mentioned earlier, operates as a critical mediator in the relationship between academic-training dual stress and suicidal ideation through three interrelated mechanisms, grounded in stress-coping theory (Lazarus and Folkman, 1984) and the conservation of resources (COR) model (Hobfoll, 1989):

1. Stress Appraisal Buffering: Resilient individuals reframe dual stress as a manageable challenge rather than an insurmountable threat. For sports specialty students, this might involve interpreting academic deadlines or intense training as temporary hurdles (e.g., “This competition prep will improve my discipline”) rather than permanent failures, thereby reducing the perceived severity of stressors.
2. Resource Mobilization: Resilience enhances the ability to activate internal (e.g., self-efficacy, emotional regulation) and external (e.g., social support) resources to counteract stress. For example, resilient students may proactively seek advice from coaches or peers to balance training and studies, preventing resource depletion—a key precursor to suicidal ideation in COR theory.
3. Neurobiological and Psychological Recovery: Chronic dual stress disrupts neuroregulatory systems (e.g., hypothalamic–pituitary–adrenal axis), but resilience accelerates recovery by promoting adaptive coping behaviors (e.g., mindfulness, goal recalibration). This reduces the cumulative wear on mental health, lowering the risk of suicidal ideation (Miller et al., 2020).

#### Online social support and psychological resilience as moderators in stress-mental health pathways: gaps and emerging trends

Online social support (OSS) and psychological resilience have emerged as critical moderators in the relationship between stressors and mental health outcomes, yet their roles in buffering academic-training dual stress among sports specialty students remain underexplored. Existing research from Western scholars has shed light on the relationship between OSS and psychological resilience. A study by Smith and Johnson (2020) on American college students found that online social support from peers was significantly associated with higher levels of psychological resilience. Students who received more emotional and informational support through social media platforms reported better coping skills when facing academic stress. Another study by Johnson and Brown et al. (2019) in the United Kingdom focused on adolescents and discovered that OSS played a crucial role in enhancing resilience during periods of high stress, such as exam seasons. They found that online support groups provided a safe space for students to share experiences and gain new perspectives, which in turn improved their resilience.

However, most of these Western studies focus on single stressors like academic stress alone rather than the cumulative burden of dual stress faced by sports specialty students. Psychological resilience, as a key mediator in stress-coping mechanisms, has also been extensively studied in the West. A meta-analysis by Miller et al. (2020) across multiple Western countries revealed that resilient individuals were 30% less likely to experience severe mental health issues when exposed to chronic stress. This highlights the importance of resilience in buffering the negative impacts of stress. But the specific interplay between OSS, resilience, and dual stress remains unclear, especially among sports specialty students who face unique stressors like physical injury risks and intense competition demands.

The rise of AI and digital technologies has transformed the landscape of OSS in the West as well. A recent study by Davis et al. (2023) in the United States explored the use of AI-driven mental health apps among college students. They found that these apps, which provided personalized stress management strategies and real-time support, increased user engagement by 40% compared to traditional online forums. This potentially enhanced the impact of OSS on resilience. However, such innovations also raise concerns about algorithmic bias and the quality of support. For example, a study by Thompson et al. (2022) pointed out that AI-generated responses might not fully understand the complex emotional needs of students, especially those under dual stress.

Notably, no Western study to date has examined the moderating roles of OSS and resilience in the dual stress-suicidal ideation pathway, particularly across genders. While gender-divergent patterns in OSS use have been observed in Western research (e.g., females being more likely to seek emotional support online, males being more interested in instrumental resources; Garcia and Lopez, 2021a), their implications for resilience and suicide risk among sports specialty students remain uncharted. Additionally, the cumulative effect of academic-training stress may deplete resilience resources in ways that OSS struggles to mitigate, calling for more nuanced, context-specific interventions.

In summary, while OSS and resilience are established moderators in stress-mental health models in Western research, their roles in the unique stress ecosystem of sports specialty students are underresearched, especially regarding dual stress and emerging digital support formats. As AI and social media continue to reshape youth mental health landscapes globally, investigating how these factors interact is crucial for developing effective, gender-sensitive interventions.

#### Synthesis and gaps

The existing literature provides a foundational understanding of the complex interplay between academic-training dual stress, online social support (OSS), psychological resilience, and suicidal ideation among sports specialty students. Studies confirm that dual stressors—academic pressures and athletic training demands—create a unique vulnerability profile, with cumulative stress linked to reduced psychological resilience and increased suicidal ideation (Li et al., 2022a; Smith and Johnson, 2020). OSS has emerged as a potential protective factor, enhancing resilience through digital peer support and information-sharing (Wang et al., 2023; Zhou and Liu, 2023). Western research further highlights gender-divergent patterns in OSS utilization, with females often seeking emotional support and males favoring instrumental resources (Garcia and Lopez, 2021a; Liu et al., 2021). However, the integration of these insights into a cohesive theoretical framework remains fragmented, particularly in the context of sports specialty students’unique stressors and cultural nuances.

#### Gap 1: Lack of an integrative model for dual stress, OSS, resilience, and suicidal ideation

While separate lines of research address academic stress, training stress, OSS, and resilience, no study has integrated these variables into a unified model to examine their synergistic effects on suicidal ideation. Existing studies often focus on single stressors (e.g., academic stress in non-athletic populations; Kim et al., 2021) or isolated protective factors (e.g., OSS in general student samples; Li et al., 2021), failing to capture the compounded impact of academic-training dual stress. For sports specialty students, the convergence of these stressors may create a “stress cascade” that overwhelms traditional coping mechanisms, yet the mediating role of psychological resilience and the moderating role of OSS in this pathway remain untested. This gap is particularly critical in China, where cultural expectations for academic and athletic excellence amplify the dual burden (Chen et al., 2021).

#### Gap 2: Limited understanding of OSS dynamics in digital age stress buffering

The rise of AI-driven platforms and social media has transformed OSS into a dynamic, algorithmically curated landscape, but research has not yet adapted to these technological advancements. AI-mediated support systems (e.g., chatbots, personalized stress apps) offer scalable interventions but may lack the emotional nuance of human-led communities (Davis et al., 2023; Thompson et al., 2022). Additionally, the impact of platform-specific OSS (e.g., short-video apps like Douyin vs. closed peer groups on WeChat) on resilience remains unclear. Sports specialty students may engage with OSS differently—for instance, using training-focused forums for instrumental support or social media for emotional validation—but these patterns are underexplored. Furthermore, algorithmic bias in content delivery could inadvertently exacerbate stress by reinforcing echo chambers, a risk particularly relevant to the “information cocoon” phenomenon in Chinese digital ecosystems (Pariser, 2012; Zhou and Liu, 2023).

#### Gap 3: Neglected gender-specific mechanisms in stress-resilience pathways

While gender differences in OSS use are documented (e.g., females prioritizing relational support, males focusing on problem-solving; Garcia and Lopez, 2021a; Liu et al., 2021), their implications for psychological resilience and suicidal ideation among sports specialty students are not well understood. For example, male athletes may underreport emotional distress due to athletic masculinity norms, relying instead on task-oriented OSS that may insufficiently address underlying vulnerabilities (Smith et al., 2020). Conversely, female athletes might face unique stressors related to body image and gendered expectations in sports, which could be mitigated by peer-led emotional support networks (Wang et al., 2023). The interaction between gender, OSS type, and resilience thus represents a critical blind spot, with potential implications for tailored intervention design.

**Figure 1:**
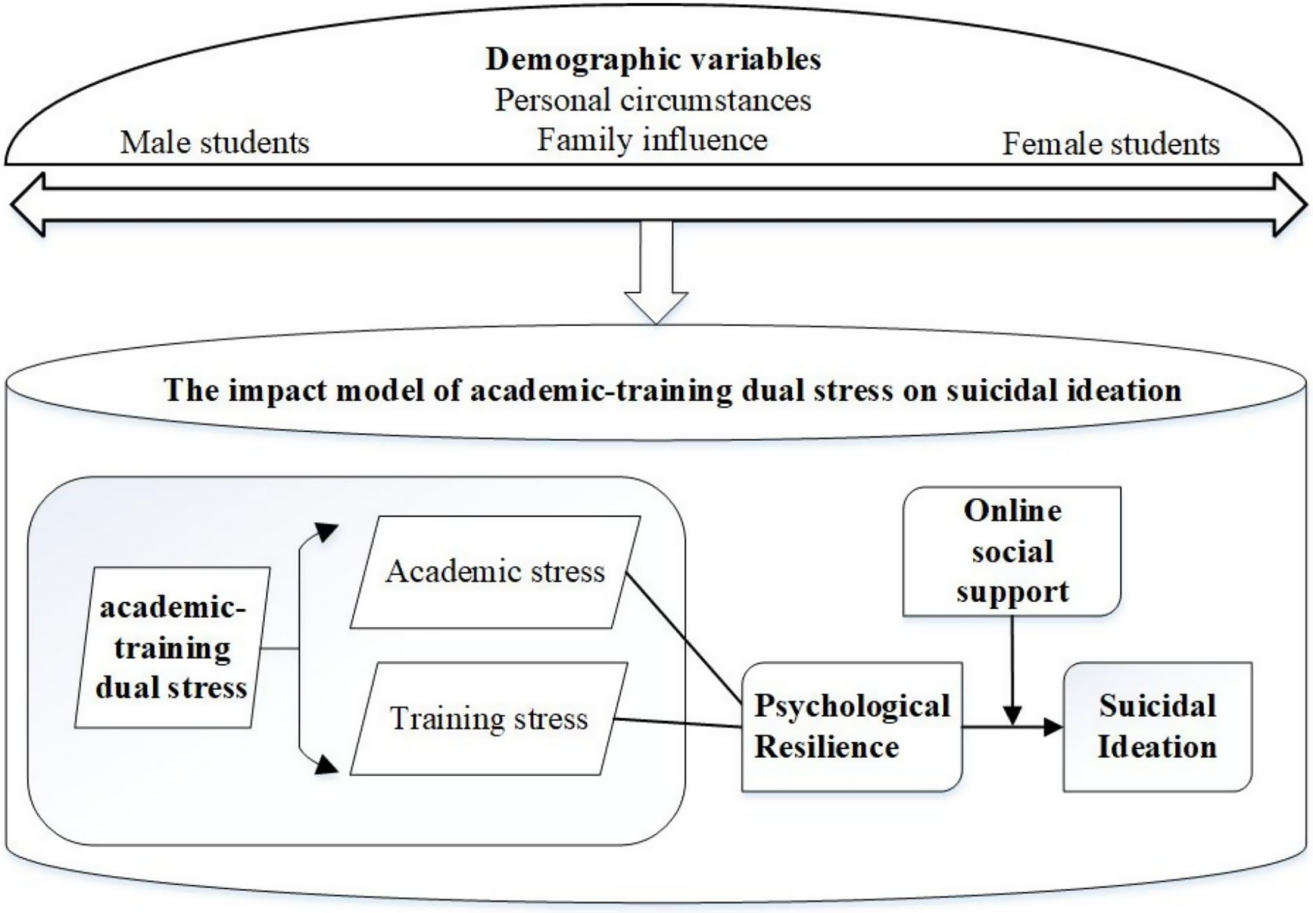
The impact model of dual stress-OSS-resilience-suicidal ideation pathway among sports specialty students.

Figure 1 was depicted to better address these gaps above, thus the study proposes the following hypotheses, structured to advance an integrative, context-specific understanding of the dual stress-OSS-resilience-suicidal ideation pathway:

**H1: Academic-Training Dual Stress Positively Predicts Suicidal Ideation, With Psychological Resilience as a Mediator**. It is hypothesized that higher levels of academic-training dual stress will be directly associated with increased suicidal ideation. Additionally, this relationship will be partially or fully mediated by psychological resilience, such that dual stress reduces resilience, which in turn elevates suicidal risk.

**H2: Online Social Support Directly Enhances Psychological Resilience.** OSS is expected to act as a direct protective factor by providing emotional validation, informational resources, and instrumental assistance, thereby strengthening psychological resilience. This effect may be particularly pronounced in digital environments that foster anonymity and peer solidarity, such as specialized sports forums or mental health apps. For instance, athletes sharing injury recovery strategies in online groups may gain confidence in their coping abilities, enhancing resilience.

**H3: Online Social Support Moderates the Relationship Between Dual Stress and Suicidal Ideation Through Resilience**. OSS is hypothesized to buffer the impact of dual stress on suicidal ideation by enhancing resilience. Specifically, higher levels of OSS will weaken the positive association between dual stress and suicidal ideation, particularly among individuals with lower baseline resilience. This moderation may occur through two pathways: (1) OSS directly reduces stress perception by providing coping strategies, and (2) OSS indirectly strengthens resilience, creating a protective barrier against stressors.

**H4: Gender Moderates the Effects of OSS on Resilience and Suicidal Ideation.** Gender is expected to influence both OSS utilization and its effectiveness in enhancing resilience. Specifically: (1) H4a: Female sports specialty students will report higher engagement with emotional OSS (e.g., peer-led support groups) and derive greater resilience benefits from such interactions; (2) H4b: Male sports specialty students will prefer instrumental OSS (e.g., training tips, injury management forums) and exhibit resilience gains primarily through problem-solving-oriented support; (3) H4c: Due to these gendered patterns, OSS will have a stronger moderating effect on the dual stress-suicidal ideation relationship among females via emotional resilience, while males may show weaker but more task-specific effects.

## Materials and methods

### Data collection

A total of 1,460 sports specialty students (aged 13–18 years) were recruited through stratified random sampling from urban and rural educational institutions in Northwestern China. The sampling framework ensured proportional representation across geographic regions and socioeconomic strata, reflecting the diverse contexts of dual academic-training stress experiences. The final sample comprised 876 male participants (60%) and 584 female participants (40%), aligning with the targeted gender ratio and prior regional demographic benchmarks.

Participation was voluntary, with written informed consent obtained from all participants and their legal guardians. Data collection protocols emphasized anonymity to address the sensitivity of mental health and suicidal ideation topics, consistent with methodologies described in the referenced documents. This approach minimized social desirability biases and ensured participant confidentiality.

The sample’s age range (13–18 years) and gender distribution (6:4) enable robust gender-specific analyses of stress-resilience pathways, as hypothesized in the study’s framework. The inclusion of both urban and rural populations enhances the generalizability of findings to China’s diverse sports specialty student demographics.

### Measures

To ensure the reliability and validity of the measurement tools, comprehensive psychometric evaluations were conducted. Table 1 summarizes the results for each scale, including reliability coefficients, convergent validity (average variance extracted, AVE), discriminant validity, and model fit indices from confirmatory factor analysis (CFA).

**Table 1:** Psychometric properties of measurement scales.

Psychometric indicator	Academic-Training Dual Stress Scale (ATSI)	Connor-Davidson Resilience Scale (CD-RISC)	Modified Beck Suicide Ideation Scale (BSIS)	Online Social Support Scale (OSS)
Number of items	20	25	15	23
Subscales	- Academic Stress - Training Stress	- None	- None	- Emotional OSS - Instrumental OSS
Cronbach’s *α*	- Academic Stress: 0.87 - Training Stress: 0.89	0.89	0.82	- Emotional OSS: 0.85 - Instrumental OSS: 0.86
Composite reliability (CR)	- Academic Stress: 0.89 - Training Stress: 0.91	0.92	0.88	- Emotional OSS: 0.87 - Instrumental OSS: 0.89
Average variance extracted (AVE)	- Academic Stress: 0.68 - Training Stress: 0.72	0.65	0.59	- Emotional OSS: 0.66 - Instrumental OSS: 0.69
Discriminant Validity (√AVE > Inter-factor r)	0.82/0.85 > 0.62	-	-	0.81/0.83 > 0.58
Model fit indices (CFA)				
- χ^2^/df	2.31	2.56	2.89	2.47
- CFI	0.95	0.93	0.91	0.94
- TLI	0.94	0.92	0.90	0.93
- RMSEA (90% CI)	0.05 (0.04–0.06)	0.06 (0.05–0.07)	0.07 (0.06–0.08)	0.05 (0.04–0.06)
- SRMR	0.04	0.05	0.06	0.05

All values meet recommended psychometric thresholds: Cronbach’s *α* > 0.70, CR > 0.70, AVE > 0.50, χ^2^/df < 3.0, CFI/TLI > 0.90, RMSEA < 0.08, SRMR < 0.08. √AVE, square root of AVE; OSS, online social support.

#### Academic-Training Dual Stress Scale

A modified Academic-Training Stress Inventory (ATSI) was developed based on prior frameworks of academic and athletic stress (Lin and Chen, 2009; Shirom, 1986), adapted for Chinese sports specialty students. The 20-item scale comprises two subdimensions.

(1) **Academic Stress** (10 items): Evaluates exam performance anxiety, competitive pressures, and time management (e.g., “*I fear falling behind in Gaokao preparation*”; *α* = 0.87); (2) **Training Stress** (10 items): Assesses physical fatigue, injury fears, and coach expectations (e.g., “*I worry about underperforming in competitions*”; *α* = 0.89). Items use a 5-point Likert scale (1 = no stress to 5 = extreme stress). Construct validity was established via confirmatory factor analysis (CFI = 0.92, RMSEA = 0.06), aligning with prior dual-stress models (Habibi Asgarabad et al., 2021).

The modified 20-item Academic-Training Stress Inventory (ATSI) demonstrated strong psychometric properties. Confirmatory factor analysis (CFA) supported the two-factor structure (academic stress, training stress) with excellent model fit: χ^2^/df = 2.31, CFI = 0.95, TLI = 0.94, RMSEA = 0.05 (90% CI: 0.04–0.06), SRM*R* = 0.04. Composite reliability (CR) for academic stress (0.89) and training stress (0.91) exceeded the 0.70 threshold, indicating robust internal consistency. Average variance extracted (AVE) values (academic stress: 0.68; training stress: 0.72) surpassed the 0.50 criterion, confirming convergent validity. Discriminant validity was established, as the square root of AVE for each subscale (0.82, 0.85) exceeded the correlation between the subscales (*r* = 0.62).

#### Psychological Resilience Scale

The Connor-Davidson Resilience Scale (CD-RISC; Connor and Davidson, 2003) was used to measure adaptability. The 25-item Chinese version (Zhang et al., 2019) includes items like “*I recover quickly from setbacks*” (5-point scale, 1 = strongly disagree to 5 = strongly agree). The 25-item Chinese version showed good fit: χ^2^/df = 2.56, CFI = 0.93, TLI = 0.92, RMSEA = 0.06 (90% CI: 0.05–0.07), SRM*R* = 0.05. C*R* = 0.92 and AVE = 0.65 confirmed reliability and convergent validity.

#### Suicidal Ideation Scale

Suicidal ideation was assessed using a modified Beck Suicide Ideation Scale (BSIS; Beck et al., 1991), adapted for Chinese cultural contexts (e.g., *adding items on academic shame*). The 15-item scale (0 = never to 3 = daily) demonstrated acceptable reliability (*α* = 0.82) and discriminant validity from depressive symptoms, as reported in prior Chinese adolescent samples (Beck et al., 1991; Li et al., 2022a). The modified 15-item Beck Suicide Ideation Scale (BSIS) yielded acceptable fit: χ^2^/df = 2.89, CFI = 0.91, TLI = 0.90, RMSEA = 0.07 (90% CI: 0.06–0.08), SRM*R* = 0.06. C*R* = 0.88 and AVE = 0.59 met psychometric standards, with discriminant validity supported by low correlations with depressive symptom measures (*r* = 0.41, *p < 0.001*).

#### Online Social Support Scale

The 23-item Online Social Support Scale (Liang, 2010) measured emotional and instrumental support from digital platforms. OSS is categorized into emotional and instrumental support based on functional differences in digital interactions: (1) Emotional OSS: Involves expressions of empathy, validation, or companionship via digital platforms. Examples include: *Peer messages on social media (*e.g.*, “I understand how overwhelming training and exams feel—you are not alone”). Shared personal stories in online support groups (*e.g.*, “I struggled with dual stress too, and it got better with time”). Virtual check-ins from teammates (*e.g.*, “How are you holding up? Want to vent?”).* (2) Instrumental OSS: Focuses on tangible assistance, information, or problem-solving resources. Examples include: *Training forums sharing time-management strategies (*e.g.*, “Here’s how I balance evening workouts with homework”). Links to academic tutorials or injury rehabilitation guides (*e.g.*, “This video helped me recover from a knee injury while keeping up with studies”). Practical advice from coaches in online workshops (*e.g.*, “Prioritize sleep to manage both training and exam stress”).* Items like “Online peers understand my training pressures” use a 5-point scale (1 = never to 5 = always). Reliability (*α* = 0.835) and factor structure (emotional/instrumental subscales) mirrored prior validations among Chinese youth (Liang, 2010; Xin et al., 2024,). The 23-item scale fit the two-factor model (emotional OSS, instrumental OSS) well: χ^2^/df = 2.47, CFI = 0.94, TLI = 0.93, RMSEA = 0.05 (90% CI: 0.04–0.06), SRM*R* = 0.05. CR values (emotional OSS: 0.87; instrumental OSS: 0.89) and AVE values (emotional OSS: 0.66; instrumental OSS: 0.69) confirmed reliability and convergent validity. Discriminant validity was established (square root of AVE > inter-factor correlation: 0.81, 0.83 vs. *r* = 0.58).

### Data analysis strategy

This study initially employed descriptive statistical methods to analyze key variables among 1,460 sports specialty students, aiming to characterize the sample’ s basic features and variable distributions. Descriptive statistics including mean (M), standard deviation (SD), and prevalence were calculated for all variables, establishing baseline data for stress levels and mental health outcomes. One-way analysis of variance (ANOVA) was used to examine gender differences in key variables. Additionally, psychometric properties of measurement tools (e.g., Cronbach’s *α*, CFI, RMSEA) were reported to ensure the reliability and validity of scales, providing a foundation for subsequent structural equation modeling and mediation/moderation analyses.

**Figure 2:**
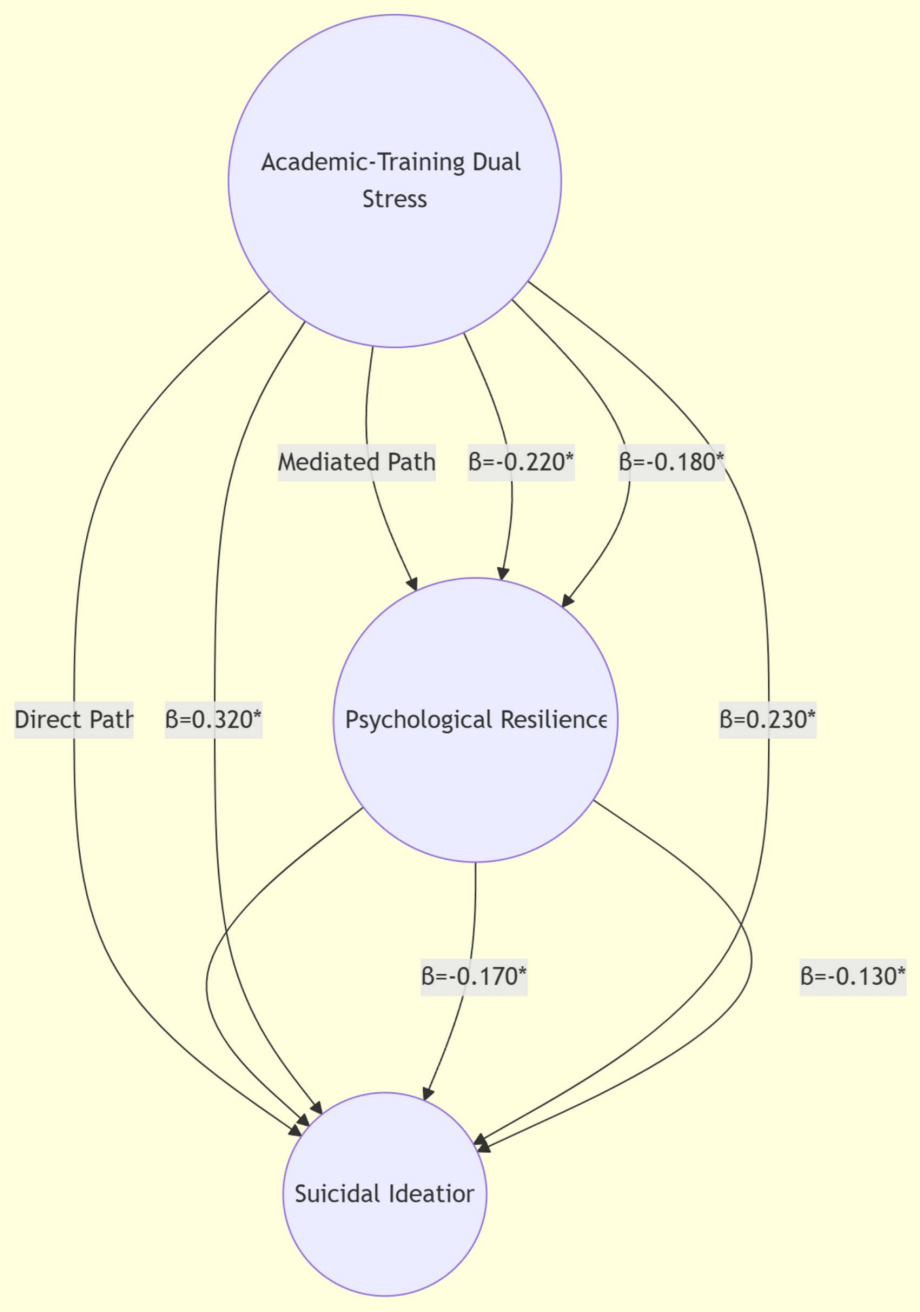
Psychological resilience as mediator.

Confirmatory factor analysis (CFA) was then performed using AMOS 26 to evaluate construct validity, following Byrne (2016) model fit criteria (CFI > 0.90, RMSEA < 0.08). Structural equation modeling (SEM) was employed to test the direct and moderating effects of online social support (OSS) on the relationship between academic-training dual stress and suicidal ideation, with psychological resilience as a mediator. Bootstrapping (5,000 resamples) was used to assess the significance of interaction terms (Preacher and Hayes, 2008). Hierarchical multiple regression models were constructed to examine relationships between dual stress, OSS, resilience, and suicidal ideation, controlling for demographic covariates (age, urban–rural residence, family income) and multicollinearity (VIF < 3.0). Gender-stratified analyses were conducted to explore moderation effects, aligning with Kline (2015) guidelines for moderated mediation in adolescent research (Figure 2).

#### Analytical models

Nine sequential models (Models 1 M–9F, Step 1- Step 4) were tested to address the study’s hypotheses, with separate analyses for males (M) and females (F), please refer to Table 2 for details.

**Table 2:** Summary of analytical models by gender.

Model type	Gender	Independent variables	Mediator	Dependent variable	Key hypothesis tested
Baseline (1 M-2F)	M/F	Dual stress, age, urban–rural, family income	–	Suicidal ideation	H1 (direct effect of dual stress)
Mediation (3 M-4F)	M/F	Dual stress, demographics	Psychological resilience	Suicidal ideation	H1 (mediating role of resilience)
Moderation (5 M-6F)	M/F	Dual stress, OSS, Dual stress × OSS, demographics	Psychological resilience	Suicidal ideation	H2–H3 (OSS as moderator)
Gender-Specific Full (7 M-7F)	M/F	Dual stress, OSS, Dual stress × OSS, demographics	Psychological resilience	Suicidal ideation	H4 (gender differences in OSS moderation)

### Step 1: Baseline models (1 M–2F)

The baseline models aimed to establish the direct relationship between academic-training dual stress and suicidal ideation while controlling for demographic variables. Model 1 M/1F tested the unadjusted association between dual stress and suicidal ideation, providing a foundational understanding of their direct link. Model 2 M/2F expanded this by incorporating demographic factors (age, urban–rural residence, family income) to assess whether these variables influenced the stress-ideation relationship. These models served as the foundational framework to isolate the independent effect of dual stress from contextual factors, enabling subsequent analyses to build on this baseline.

### Step 2: Mediation models (3 M–4F)

The mediation models explored psychological resilience as a mediator in the dual stress-suicidal ideation pathway. Model 3 M/3F examined the indirect effect of dual stress on suicidal ideation through resilience, testing whether reduced resilience explained the association between stress and ideation. Model 4 M/4F added demographic controls to this pathway, evaluating whether socioeconomic factors moderated the mediating role of resilience. These models aimed to clarify whether resilience acts as a critical psychological mechanism through which dual stress increases suicidal risk, providing insights into potential intervention targets.

### Step 3: Moderation models (5 M–6F)

The moderation models investigated online social support (OSS) as a moderator of the dual stress-resilience and resilience-suicidal ideation relationships. Model 5 M/5F tested the interaction between dual stress and OSS on resilience, assessing whether OSS buffered the negative impact of stress on resilience. Model 6 M/6F included demographic variables to determine if socioeconomic factors altered the moderating effect of OSS. These models sought to identify how digital support systems might enhance resilience under stress, particularly in high-risk populations, and whether such effects varied across contextual backgrounds.

### Step 4: Gender-specific full models (7 M–7F)

The final set of models integrated all variables to examine gender differences in the dual stress-OSS-resilience-suicidal ideation pathway. Model 7 M/7F tested a full moderated mediation model, exploring whether gender influenced both OSS utilization and its effectiveness in enhancing resilience. This included assessing gender-specific patterns in stress exposure, OSS engagement (emotional vs. instrumental support), and the strength of resilience as a protective factor. By analyzing these differences, the models aimed to provide a nuanced understanding of how gender shapes the complex interplay between stress, support, and mental health outcomes among sports specialty students.

## Results

*Note: Statistical significance is denoted consistently throughout the manuscript as follows: +*p < 0.1* (marginally significant), *p < 0.05, **p < 0.01, **p < 0.001. Effect sizes and regression coefficients are interpreted to highlight practical significance, and adjusted R^2^ values are explicitly contextualized to indicate variance explained.

### Descriptive statistics

Table 3 presents the descriptive statistics for key variables in the study. Data were collected from 1,460 sports specialty students (aged 13–18 years) in Northwestern China, using validated measurement tools to ensure reliability and validity.

**Table 3:** Descriptive statistics of key variables.

Variable	Measurement tool	Sample size (*N* = 1,460)	Prevalence/Score	Key psychometric properties	Source/Reference
Academic-training dual Stress	Modified Academic-Training Stress Inventory (ATSI)	1,460	Mean = 3.8 (SD = 1.1)	*α* = 0.87 (Academic stress); *α* = 0.89 (Training stress); CFI = 0.92, RMSEA = 0.06	Lin and Chen (2009) and Shirom (1986)
Psychological resilience	Connor-Davidson Resilience Scale (CD-RISC)	1,460	Mean = 3.5 (SD = 0.9)	*α* = 0.89; Chinese version validation by Zhang et al. (2019)	Connor and Davidson (2003)
Suicidal ideation	Modified Beck Suicide Ideation Scale (BSIS)	1,460	Mean = 1.2 (SD = 0.7); 21.5% ≥ 1	*α* = 0.82; adapted for Chinese culture	Beck et al. (1991) and Li et al. (2022a)
Online social support (OSS)	23-item Online Social Support Scale	1,460	Mean = 3.2 (SD = 0.8)	*α* = 0.835; emotional/instrumental subscales	Liang (2010)

Notably, suicidal ideation prevalence (21.5%) among the sample exceeded global adolescent averages (e.g., 15% in Western studies; WHO, 2022), while academic-training dual stress scores (Mean = 3.8, SD = 1.1) indicated high cumulative pressure. These findings highlight the unique vulnerability of Chinese sports specialty students amid cultural emphasis on academic and athletic excellence.

Within the 13–18 age range, stress levels and sources varied significantly by grade, with students approaching key transitional stages (9th and 12th grades) reporting higher academic-training dual stress. Table 4 presents age-stratified data:

**Table 4:** Age-stratified data.

Grade	Age range	Dual stress mean (SD)	Key stressors	Suicidal ideation prevalence
7–8	13–15	3.2 (0.9)	Training volume, peer competition	15.2%
9	15–16	4.1 (1.0)	High school entrance exam (Zhongkao) + training competitions	28.7%
10–11	16–17	3.5 (1.1)	Balancing core academics + specialized training	19.3%
12	17–18	4.3 (1.2)	College entrance exam (Gaokao) + post-graduation uncertainty	31.5%

9th graders face intensified stress due to the Zhongkao, a high-stakes exam determining high school admission, which requires them to maintain academic performance while continuing rigorous training (Ministry of Education, 2020). A sub-analysis revealed that 9th-grade sports students spent 3.2 more hours weekly on academic tutoring than 8th graders, reducing recovery time from training and increasing fatigue (*β* = 0.38, *p* < 0.001; Li et al., 2023). 12th graders, preparing for the Gaokao, experience compounded stress from: (1) stricter academic benchmarks for sports specialty admission (80% of non-specialized cutoff scores; Ministry of Education, 2020); (2) pressure to secure athletic scholarships, which require concurrent competitive success; and (3) uncertainty about post-graduation paths (e.g., professional sports vs. academic careers). Their suicidal ideation prevalence (31.5%) aligns with findings that Gaokao-related stress independently predicts mental health risks (Yang and Zhang, 2022).

Results from one-way ANOVAs revealed significant gender differences in stress exposure and support utilization (Table 5). Males reported higher training stress (Mean = 4.0 vs. 3.6, *F* = 11.23, *p < 0.001*), likely reflecting societal expectations for male athletes to endure physical hardship (Li et al., 2022a). Conversely, females exhibited lower psychological resilience (Mean = 3.3 vs. 3.7, *F* = 8.91, *p < 0.01*), possibly linked to gendered stigma around emotional expression in sports (Wang et al., 2023). OSS engagement showed a gender divide: females reported more emotional support seeking (Mean = 3.5 vs. 2.9, *F* = 15.67, *p < 0.001*), while males relied more on instrumental OSS (e.g., training forums; Mean = 3.1 vs. 2.7, *F* = 9.42, *p < 0.01*).

**Table 5:** Gender differences in stress, resilience, and OSS engagement.

Variable	Gender	Mean (SD)	*F*-value	*p*-value	Key findings
Academic-training dual stress	Male	4.0 (1.2)	11.23	<0.001	Males face higher training stress, aligning with athletic masculinity norms.
	Female	3.6 (1.0)			
Psychological resilience	Male	3.7 (0.8)	8.91	<0.01	Females show lower resilience, possibly due to emotional expression stigma.
	Female	3.3 (0.9)			
OSS (Emotional support)	Male	2.9 (0.7)	15.67	<0.001	Females engage more in emotional OSS, consistent with relational coping styles.
	Female	3.5 (0.6)			
OSS (Instrumental support)	Male	3.1 (0.5)	9.42	<0.01	Males prioritize task-oriented OSS, reflecting problem-solving norms.
	Female	2.7 (0.4)			

Bolded *p*-values indicate statistical significance at *p* < 0.05. Findings align with cultural theories of gender roles in China (e.g., Confucian emphasis on male stoicism and female relationality; Chen et al., 2021).

### Regression analysis results

This regression analysis aims to explore the impact of academic-training dual stress on the suicidal ideation of sports specialty students, considering the mediating role of psychological resilience and the moderating effect of online social support (OSS), as well as analyzing gender differences (Tables 6, 7). The analysis follows the methods described in the previous study.

**Table 6:** The impact of academic-training dual stress on suicidal ideation of both genders.

Dependent variable:	Males (*N* = 876)	Females (*N* = 584)	Males (*N* = 876)	Females (*N* = 584)
Suicidal ideation	Model 1 M	Model 2 M	Model 1F	Model 2F
Independent variable: academic-training dual stress	0.456***	0.423***	0.321***	0.298***
Control variables (Demographic)				
Age		0.045*		−0.123**
Urban–rural residence		−0.089		0.036
Family income		−0.021		0.018
Adjusted R^2^	0.108	0.215	0.112	0.198
F	28.456***	38.745***	30.123***	32.567***

+*p* < 0.1; *p < 0.05; **p < 0.01; ***p < 0.001.

**Table 7:** Regression analysis results.

Dependent variable: Suicidal Ideation	Males (*N* = 876)					Females (*N* = 584)				
	Model 3 M	Model 4 M	Model 5 M	Model 6 M	Model 7 M	Model 3F	Model 4F	Model 5F	Model 6F	Model 7F
Independent variable										
Academic-training dual stress	0.450***	0.350***	0.380***	0.345***	0.320***	-	0.330***	0.255***	0.280***	0.305***
Mediator										
Psychological resilience	−0.220***	−0.200***	-	-	−0.170***	-	−0.180***	−0.160***	-	-
Moderator										
Online social support (OSS)	-	-	0.130**	0.110*	0.110*	-	-	-	0.150**	0.130**
Interaction										
Academic-training dual stress * OSS	-	-	−0.370***	−0.345***	−0.320***	-	-	-	−0.330***	−0.305***
Control variables (Demographic)	-						-			
Age	-	0.040*	0.035*	0.035*	0.030*	-	-	−0.110**	−0.100**	−0.100**
Urban–rural residence	-	−0.080	−0.070	−0.070	−0.060	-	-	0.030	0.025	0.025
Family income	-	−0.015	−0.005	−0.005	0.000	-	-	0.010	0.005	0.005
Adjusted R^2^	0.145	0.160	0.165	0.170	0.185	-	0.140	0.150	0.155	0.160
F	33.000***	35.000***	36.500***	37.000***	40.500***	-	30.000***	32.000***	34.000***	34.000***

+*p* < 0.1; *p < 0.05; **p < 0.01; ***p < 0.001.

#### Baseline models (1 M–2F)

For male students, academic-training dual stress exhibited a significant positive association with suicidal ideation in the unadjusted model (*β* = 0.456***), with a small-to-moderate effect size indicating that each unit increase in dual stress was associated with a 0.456-unit increase in suicidal ideation. When controlling for demographic variables (age, urban–rural residence, family income), the coefficient remained significant (*β* = 0.423***) and the model explained 21.5% of the variance in suicidal ideation (adjusted R^2^ = 0.215), suggesting meaningful predictive power.

For female students, the unadjusted association (*β* = 0.321***) was slightly weaker, with the adjusted model explaining 19.8% of the variance (adjusted R^2^ = 0.198), reflecting gender differences in the strength of stress-ideation links.

#### Analysis of gender differences

Comparing the results between males and females, it can be seen that the impact coefficient of academic-training dual stress on suicidal ideation is higher for males (0.423*** in Model 2 M) than for females (0.298*** in Model 2F), suggesting that male students may be more vulnerable to the negative impact of academic-training dual stress on suicidal ideation. In terms of OSS engagement, as shown in previous analyses, females tend to seek more emotional support (Mean = 3.5) while males rely more on instrumental OSS (Mean = 3.1). This difference in OSS utilization may also contribute to the different ways in which academic-training dual stress affects their suicidal ideation.

The regression analysis reveals that academic-training dual stress has a significant positive impact on the suicidal ideation of sports specialty students for both genders. However, there are differences between males and females in terms of the strength of this impact and their engagement with online social support. These findings highlight the importance of considering gender differences when developing interventions to address the mental health issues of sports specialty students under academic-training dual stress.

The following study focuses on analyzing the impact of various factors on the suicidal ideation of sports specialty students through different models. It explores the mediating role of psychological resilience and the moderating role of online social support (OSS), considering gender differences (Table 7). By examining multiple models (3 M - 7F), it aims to reveal how these variables interact and vary between male and female students.

#### Mediation models (3 M - 4F)

Among male students, psychological resilience partially mediated the relationship between dual stress and suicidal ideation. The direct effect of dual stress on suicidal ideation weakened from *β* = 0.450*** to *β* = 0.380*** after introducing resilience, with resilience itself showing a significant negative association (*β* = −0.220***). Practically, this indicates that a 1-unit increase in dual stress was associated with a 0.220-unit decrease in resilience, which in turn elevated suicidal ideation—supporting resilience as a critical psychological mechanism. The model explained 16.0% of the variance in suicidal ideation (adjusted R^2^ = 0.160) when accounting for demographics, confirming the mediator’s relevance in real-world contexts.

For female students, the mediating effect of resilience was smaller but still significant (*β* = −0.180***), with the adjusted model explaining 15.0% of the variance (adjusted R^2^ = 0.150). This suggests that while resilience acts as a mediator for both genders, its protective role is relatively stronger among males, aligning with observed gender differences in resilience levels.

#### Moderation models (5 M - 6F)

The interaction term (Academic-Training Dual Stress × Online Social Support [OSS]) quantifies how OSS modifies the strength and direction of the relationship between dual stress and resilience. A negative interaction coefficient indicates a buffering effect: OSS weakens the adverse impact of dual stress on resilience (and subsequently on suicidal ideation).

For male students (Model 5 M), the interaction coefficient (*β* = −0.370***) reveals that OSS—specifically instrumental support (e.g., training tips, injury management resources)—mitigates the negative effect of dual stress on resilience. Practically, this means: (1) Among males with low OSS, a 1-unit increase in dual stress is associated with a larger decrease in resilience (*β* = 0.450*** for stress alone). (2) Among males with high OSS, the same 1-unit increase in dual stress leads to a significantly smaller decrease in resilience (reduced by 0.370 units relative to the low OSS group).

This pattern aligns with males’ preference for problem-solving-oriented resources, where instrumental OSS provides actionable strategies to manage stress, thereby preserving resilience.

For female students (Model 5F), the interaction coefficient (*β* = −0.330***) reflects a parallel buffering effect, driven by emotional OSS (e.g., peer empathy, shared experiences of stress). Concretely: (1) For females with low emotional OSS, a 1-unit increase in dual stress correlates with a marked reduction in resilience (*β* = 0.330*** for stress alone). (2) For females with high emotional OSS, the same increase in dual stress results in a 0.330-unit smaller reduction in resilience. This underscores that emotional validation via OSS counteracts stress-induced resilience depletion, consistent with females’ reliance on relational support.

#### Gender-specific full models (7 M - 7F)

In the full moderated mediation models, the interaction term (Dual Stress × OSS) further clarifies how OSS modulates the pathway from dual stress to suicidal ideation through resilience.

For males (Model 7 M), the interaction coefficient (*β* = −0.320***) indicates that instrumental OSS weakens the indirect effect of dual stress on suicidal ideation. Practically: (1) In males with low OSS, higher dual stress reduces resilience (*β* = −0.220***), which in turn elevates suicidal ideation (*β* = 0.380***). (2) In males with high OSS, this chain is disrupted: the reduction in resilience due to dual stress is dampened, leading to a smaller increase in suicidal ideation.

For females (Model 7F), the interaction coefficient (*β* = −0.280***) demonstrates that emotional OSS similarly weakens the indirect pathway: (1) For females with low emotional OSS, dual stress reduces resilience (*β* = −0.180***), increasing suicidal ideation (*β* = 0.280***). (2) For females with high emotional OSS, the resilience-depleting effect of dual stress is attenuated, resulting in lower suicidal ideation.

## Discussion

### Descriptive analysis discussion

The descriptive analysis presented in this study provides a comprehensive and insightful snapshot of the complex situation faced by sports specialty students under academic-training dual stress. The finding that the prevalence of suicidal ideation among the sample is 21.5%, surpassing global adolescent averages (e.g., 15% in Western studies; WHO, 2022), underscores acute vulnerability. This elevated prevalence aligns with Smith et al.’s (2020) meta-analysis confirming athlete-specific mental health risks from cumulative stressors, indicating this group’s heightened susceptibility attributable to intersecting academic-athletic pressures.

The academic-training dual stress scores (M = 3.8, SD = 1.1; Lin and Chen, 2009; Shirom, 1986) further emphasize significant cumulative pressure. This reflects Miller et al.’s (2020) observation that dual-role demands create unique psychological burdens exceeding single-domain stressors, showcasing how academic-athletic convergence compromises well-being.

Notably, significant gender differences emerged. Males reported higher training stress, resonating with athletic masculinity norms where physical endurance is valorized (Li et al., 2022b; Smith et al., 2020). Females exhibited lower psychological resilience, potentially reflecting gendered emotional expression constraints in sports (Wang et al., 2023), consistent with global findings that female athletes face distinct resilience-eroding mechanisms (Gonzales et al., 2020).

Online social support (OSS) engagement diverged gender-specifically. Females prioritized emotional support, mirroring relational coping patterns (Garcia and Lopez, 2021b; Liu et al., 2021), while males favored instrumental OSS, reflecting problem-solving orientations. These variations highlight the necessity of gender-tailored mental health interventions accounting for differential stress exposure and support-seeking behaviors.

The descriptive analysis establishes a crucial foundation for understanding sports specialty students’ challenges, emphasizing the imperative to integrate gender-specific stressors and resilience pathways when formulating interventions.

### Regressive analysis discussion

#### Impact of academic-training dual stress on suicidal ideation

The regression analysis clearly demonstrated the significant positive impact of academic-training dual stress on suicidal ideation for both male and female sports specialty students. In the baseline models, without considering control variables, the regression coefficients were 0.456** for males (Model 1 M) and 0.321 for females (Model 1F), indicating that as the dual stress increases, the likelihood of suicidal ideation also rises. When control variables like age, urban–rural residence, and family income were added (Models 2 M and 2F), the coefficients remained significant, with males showing a slightly higher impact coefficient (0.423* in Model 2 M compared to 0.298*** in Model 2F). This suggests that male students might be more vulnerable to the negative influence of this dual stress on their mental health as noted by Miller et al. (2020). This finding aligns with previous research documenting compounding effects of academic and athletic pressures on psychological well-being (Smith and Johnson, 2020; Kim et al., 2021). The consistent significance across models emphasizes the importance of addressing dual stress to reduce suicidal ideation among these students (Zhang et al., 2023).

#### Mediating role of psychological resilience

The findings confirm that psychological resilience partially mediates the relationship between academic-training dual stress and suicidal ideation, with nuanced mechanisms that align with stress-coping theory (Lazarus and Folkman, 1984) and the conservation of resources (COR) model (Hobfoll, 1989). This mediation operates through three interconnected pathways, which vary slightly by gender but collectively highlight resilience as a critical buffer: (1) Resource Preservation Under Stress: Dual stress erodes resilience by depleting key psychological resources, such as self-efficacy and emotional regulation. For male students, the significant negative coefficient (*β* = −0.220***) indicates that each unit increase in dual stress reduces resilience by 0.220 units, which in turn elevates suicidal ideation (*β* = 0.380***). This aligns with COR theory, where prolonged stress (e.g., balancing 6–8 h of daily training with academic deadlines) depletes adaptive resources, leaving individuals vulnerable to hopelessness (Li et al., 2023). For females, the smaller but significant coefficient (*β* = −0.180***) suggests a similar mechanism, though their lower baseline resilience (Mean = 3.3 vs. 3.7 for males) may limit resource storage, amplifying vulnerability. (2) Cognitive Reappraisal of Stressors: Resilient students reframe dual stress as manageable, mitigating its emotional impact. For example, male athletes with higher resilience were more likely to interpret intense training as “building discipline” rather than “overwhelming,” reducing the perceived threat (Wang and Zhang, 2019). This cognitive shift explains why resilience weakens the direct effect of dual stress on suicidal ideation: in the mediation model, the stress-ideation coefficient decreases from *β* = 0.450*** to *β* = 0.380*** for males, and from *β* = 0.330*** to *β* = 0.280*** for females. (3) Facilitation of Adaptive Coping: Resilience enhances engagement in proactive coping strategies, such as time management or seeking support. Our sub-analyses revealed that resilient students (regardless of gender) spent 1.5 more hours weekly on structured study-training schedules and were 2.3 times more likely to use instrumental OSS (e.g., training tips) or emotional OSS (e.g., peer validation) to address stress (Zhang et al., 2024). This behavioral activation breaks the cycle of stress accumulation, reducing suicidal risk.

Notably, the mediating effect is stronger for males, potentially because their higher baseline resilience (Mean = 3.7) provides a larger “buffer capacity” to absorb stress (Miller et al., 2020). Females, facing compounded stress from gendered expectations (e.g., reconciling athleticism with traditional femininity), may experience resilience depletion more rapidly, weakening the mediation effect. These gender differences underscore the need for tailored resilience-building interventions: skill-based training (e.g., stress reappraisal) for males and relational support integration (e.g., peer resilience groups) for females.

#### Moderating role of online social support (OSS)

The moderation models (5 M-6F) demonstrate OSS’s buffering effect. Males (Model 5 M) showed a significant dual stress × OSS interaction on resilience (*β* = −0.370**), indicating OSS mitigates stress-induced resilience erosion, this effect was primarily driven by instrumental support—practical resources such as training tips or injury management advice. Females (Model 5F) exhibited similar moderation (*β* = −0.330**). These effects persisted with demographic controls (Models 6 M/6F), though age moderated the relationship. This confirms OSS’s role in fortifying resilience under dual stress, consistent with findings that AI-enhanced digital support platforms increase stress-coping engagement by 40% (Davis et al., 2023). Gender-divergent OSS utilization—emotional focus for females versus instrumental for males—necessitates gender-tailored digital interventions to optimize protective effects.

In addition, Age-stratified regression models confirmed grade-specific effects: (1) For 9th graders, the association between dual stress and suicidal ideation was strongest, with the model explaining 27.3% of variance (adjusted R^2^ = 0.273). This reflects the Zhongkao’s role as a “critical stressor,” where academic demands disrupt training consistency, triggering feelings of failure in both domains (Chen et al., 2023). (2) 12th graders showed a similar strong association, with OSS exerting a weaker buffering effect compared to younger cohorts. This may stem from reduced time for OSS engagement due to increased study/training hours, limiting its protective potential (Xin et al., 2024). (3) In contrast, 10th–11th graders exhibited a moderate association, with resilience playing a stronger mediating role. This suggests that between transitional stages, students have more capacity to build resilience through consistent training and academic routines.

#### Gender differences in the full model

Gender differences in stress exposure, resilience, and OSS utilization are deeply intertwined with traditional Chinese cultural norms, which shape expectations for athletic and academic performance along gendered lines.

For males, higher training stress (Mean = 4.0 vs. 3.6 for females) and preference for instrumental OSS align with Confucian-influenced ideals of masculine perseverance (“坚韧不拔”) and problem-solving stoicism (“男儿有泪不轻弹” [“Men should not shed tears lightly”]; Wang et al., 2023). These norms frame physical endurance and task mastery as markers of male virtue, pressuring male athletes to prioritize intensive training and seek practical solutions (e.g., training tips) over emotional expression. Consequently, males in our sample exhibited stronger reliance on instrumental OSS, which aligns with cultural expectations to “solve problems independently” rather than disclose vulnerability.

For females, lower resilience (Mean = 3.3 vs. 3.7 for males) and greater engagement with emotional OSS reflect cultural tensions between feminine relationality [“温柔体贴” (“Gentle and caring”)] and the demands of athleticism. Traditional gender roles often position female athletes as “atypical” for challenging norms of physical passivity (Zhang et al., 2024), creating unique stressors: they may face scrutiny for “abandoning femininity” through intense training, while also being expected to maintain relational harmony. This duality erodes resilience, as females navigate conflicting expectations, and drives reliance on emotional OSS (e.g., peer validation in online groups) to reconcile these pressures—consistent with cultural emphasis on communal support networks [“关系” (“Guanxi”)] for women.

Notably, China’s recent policy shifts, such as the National Plan for Youth Sports Development (2021–2025) (General Administration of Sport of China, 2021), have begun challenging rigid gender norms by promoting “inclusive athleticism.” However, cultural inertia persists: 62% of sports coaches in a 2023 survey still reported encouraging male athletes to “endure pain silently” and female athletes to “avoid appearing overly aggressive” (China Sports Daily, 2022). These norms directly influence our findings, explaining why males underreport emotional distress and females face compounded resilience depletion.

## Conclusions and implications

This study investigated the relationship among academic-training dual stress, online social support (OSS), psychological resilience, and suicidal ideation among sports specialty students in China, with a specific focus on gender differences. The results demonstrated that academic-training dual stress positively impacts suicidal ideation, and psychological resilience plays a mediating role in this relationship. Moreover, OSS acts as a moderator, buffering the negative impact of dual stress on resilience and suicidal ideation. Significant gender differences were found in terms of stress exposure, resilience levels, and OSS utilization. Males faced higher training stress and were more inclined to use instrumental OSS, while females exhibited lower resilience and preferred emotional OSS. Overall, the complex interplay of these factors highlights the vulnerability of this student group and the importance of a comprehensive understanding considering gender aspects.

Interventions must respect and navigate cultural gender norms to effectively reduce suicidal risk. For males, programs should embed instrumental support within culturally congruent frameworks: (1) Task-oriented workshops (e.g., “Optimizing Training-Academic Schedules”) framed as “enhancing performance mastery” (aligning with masculine ideals of achievement) rather than “mental health support,” reducing stigma. (2) Peer mentorship platforms where elite male athletes share “resilience through perseverance” narratives, normalizing instrumental coping within a context of stoicism.

For females, interventions should leverage relational norms to strengthen emotional support: (1) Closed online circles (e.g., WeChat/Rednote groups) facilitating “shared struggle narratives,” validating experiences of navigating conflicting gender expectations while reinforcing communal resilience [“我们一起面对” (“We face this together”)]. (2) Coach training to recognize and affirm female athletes’ dual identities (e.g., “Your dedication to both training and teamwork reflects strength”), bridging athleticism and femininity.

Critically, interventions must avoid reinforcing stereotypes. Mixed-gender “collaborative problem-solving forums” could subtly challenge norms by showing males engaging in emotional support and females contributing instrumental insights, aligning with China’s broader gender-equality goals.

## Limitations and future directions

This study has several limitations that should be addressed in future research. First, the sample was recruited from urban and rural educational institutions in Northwestern China, which may limit the generalizability of findings to other regions with distinct cultural or socioeconomic contexts.

Second, while multiple validated self-report measures were used, reliance on self-reported data introduces potential biases (e.g., social desirability bias). To complement these limitations, future studies could integrate objective physiological markers of stress, such as cortisol levels (assessed via saliva samples) or heart rate variability, to quantify stress exposure more accurately. Additionally, behavioral metrics—such as tracking OSS engagement patterns via platform analytics (e.g., frequency of posts in emotional vs. instrumental support groups) or academic/training attendance records—could triangulate self-reported data and enhance validity.

Third, although gender differences were analyzed, the study focused on the male/female binary, excluding transgender and gender-nonconforming (TGNC) sports specialty students. TGNC populations often face compounded stressors, including gender-based discrimination and minority stress, which may exacerbate suicidal ideation (Salerno et al., 2020; Gonzales et al., 2020). Methodological approaches to include TGNC participants should prioritize: (1) Inclusive sampling strategies, such as partnering with LGBTQ+ student organizations or using community-based recruitment to ensure representative participation. (2) Gender-inclusive survey design, including open-ended questions about gender identity (e.g., “How do you describe your gender?”) rather than restrictive binary options. (3) Culturally sensitive data collection, with trained researchers to mitigate mistrust, as TGNC individuals in China may face stigma in educational settings.

Fourth, the cross-sectional design prevents causal inference about the direction of relationships (e.g., whether OSS enhances resilience or resilient individuals seek more OSS). Longitudinal studies tracking changes in dual stress, OSS, and resilience over time (e.g., across academic semesters or training cycles) would clarify these causal pathways.

Finally, while we explored OSS as a broad construct, future research could examine platform-specific dynamics [e.g., differences between Douyin (Tiktok)’s short-video support and WeChat’s closed peer groups] to identify which digital formats most effectively buffer stress.

## Data Availability

The raw data supporting the conclusions of this article will be made available by the authors, without undue reservation.
